# Development of expressed sequence tag and expressed sequence tag–simple sequence repeat marker resources for *Musa acuminata*

**DOI:** 10.1093/aobpla/pls030

**Published:** 2012-11-26

**Authors:** Marco A. N. Passos, Viviane de Oliveira Cruz, Flavia L. Emediato, Cristiane de Camargo Teixeira, Manoel T. Souza, Takashi Matsumoto, Vânia C. Rennó Azevedo, Claudia F. Ferreira, Edson P. Amorim, Lucio Flavio de Alencar Figueiredo, Natalia F. Martins, Maria de Jesus Barbosa Cavalcante, Franc-Christophe Baurens, Orzenil Bonfim da Silva, Georgios J. Pappas, Luc Pignolet, Catherine Abadie, Ana Y. Ciampi, Pietro Piffanelli, Robert N. G. Miller

**Affiliations:** 1 Universidade de Brasília, Campus Universitário Darcy Ribeiro, Instituto de Ciências Biológicas, Asa Norte, CEP 70910-900, Brasília, DF, Brazil; 2 Postgraduate Program in Genomic Science and Biotechnology, Universidade Católica de Brasília, SGAN 916, Módulo B, CEP 70.790-160, Brasília, DF, Brazil; 3 EMBRAPA Recursos Genéticos e Biotecnologia, Parque Estação Biológica, CP 02372, CEP 70.770-900, Brasília, DF, Brazil; 4 National Institute of Agrobiological Resources, Tsukuba 305-8602, Japan; 5 EMBRAPA Mandioca e Fruticultura Tropical, Rua Embrapa, CEP 44380-000, Cruz das Almas, BA, Brazil; 6 EMBRAPA Acre, CP 321 BR 364 Km 14, Rio Branco, AC, Brazil; 7 CIRAD/UMR DAP 1098, TA A 96/03 Avenue Agropolis, 34098 Montpellier Cedex 5, France; 8 CIRAD/UMR BGPI, TA A 54/K Campus International de Baillarguet, 34398 Montpellier Cedex 5, France; 9 Present address: Genomics Platform at Parco Tecnologico Padano, Via Einstein, Località Cascina Codazza, 26900 Lodi, Italy

## Abstract

**Background and aims:**

Banana (*Musa acuminata*) is a crop contributing to global food security. Many varieties lack resistance to biotic stresses, due to sterility and narrow genetic background. The objective of this study was to develop an expressed sequence tag (EST) database of transcripts expressed during compatible and incompatible banana–*Mycosphaerella fijiensis* (*Mf*) interactions. Black leaf streak disease (BLSD), caused by *Mf*, is a destructive disease of banana. Microsatellite markers were developed as a resource for crop improvement.

**Methodology:**

cDNA libraries were constructed from *in vitro*-infected leaves from BLSD-resistant *M. acuminata* ssp. *burmaniccoides* Calcutta 4 (MAC4) and susceptible *M. acuminata* cv. Cavendish Grande Naine (MACV). Clones were 5′-end Sanger sequenced, ESTs assembled with TGICL and unigenes annotated using BLAST, Blast2GO and InterProScan. Mreps was used to screen for simple sequence repeats (SSRs), with markers evaluated for polymorphism using 20 diploid (AA) *M. acuminata* accessions contrasting in resistance to *Mycosphaerella* leaf spot diseases.

**Principal results:**

A total of 9333 high-quality ESTs were obtained for MAC4 and 3964 for MACV, which assembled into 3995 unigenes. Of these, 2592 displayed homology to genes encoding proteins with known or putative function, and 266 to genes encoding proteins with unknown function. Gene ontology (GO) classification identified 543 GO terms, 2300 unigenes were assigned to EuKaryotic orthologous group categories and 312 mapped to Kyoto Encyclopedia of Genes and Genomes pathways. A total of 624 SSR loci were identified, with trinucleotide repeat motifs the most abundant in MAC4 (54.1 %) and MACV (57.6 %). Polymorphism across *M. acuminata* accessions was observed with 75 markers. Alleles per polymorphic locus ranged from 2 to 8, totalling 289. The polymorphism information content ranged from 0.08 to 0.81.

**Conclusions:**

This EST collection offers a resource for studying functional genes, including transcripts expressed in banana–*Mf* interactions. Markers are applicable for genetic mapping, diversity characterization and marker-assisted breeding.

## Abstract (precis)

Many varieties of banana (*Musa acuminata*) lack resistance to biotic stresses. An EST collection was developed, including transcripts expressed in banana-*Mycosphaerella fijiensis* interactions. Developed polymorphic gene-derived SSR markers are applicable for genetic mapping, diversity characterization and marker assisted breeding.

## Introduction

Commercially cultivated varieties of banana and plantains are derived from the progenitors *Musa acuminata* Colla (AA) and *Musa balbisiana* Colla (BB). These crops are of extreme importance across the world's tropical and sub-tropical regions, contributing to both food security and export commodity revenue, with a global annual production in excess of 97 million tonnes (FAOSTAT 2009).

Cultivated bananas have evolved from hybridization of wild species of *M. acuminata* (A genome) and *M. balbisiana* (B genome). In contrast to fertility in wild species, many of today's commercial cultivars are sterile triploids or diploids, with fruit development via parthenocarpy. Together with female sterility, this results in either seedless fruits or non-viable seeds. Consequential asexually driven evolution has resulted in a narrow genetic base, with the crop often lacking resistance to pests and diseases. For this reason, the industry has witnessed numerous pathogen and pest outbreaks. Of the >40 fungal diseases affecting banana (Jones 1999), the foliar pathogen *Mycosphaerella fijiensis* (*Mf*) is today one of the most threatening. Responsible for black leaf streak disease (BLSD) in banana, commonly known as black Sigatoka, yield losses range from 20 to 80 % (Churchill 2011), with premature fruit ripening also affecting export markets. Although cultural practices contribute to disease control, without the integrated use of chemicals their impact is insufficient. Commercial banana plantations are therefore dependent upon long-term use of agrochemicals, which implies a constant threat for the emergence of fungicide-tolerant or -resistant *Mf* strains. The development of disease-resistant genotypes is today therefore regarded as the most cost-effective long-term control strategy available for the *Musa* industry.

Current breeding strategies for *Musa* rely upon sexually active wild or improved fertile *M. acuminata* diploids, which, in contrast to most commercial *Musa* varieties, where genetic diversity is fixed by vegetative propagation, serve as sources of resistance to biotic and abiotic stresses for transfer across varieties. Programmes for the development of tetraploid hybrids, for example, are typically generated via crosses between semi-fertile established triploids and wild or improved fertile diploid parents with agronomic traits of interest (Ortiz 1997; Amorim *et al.* 2011). Such breeding strategies can, however, have only limited success, given low numbers or absence of seeds. Complementary strategies for resolving these constraints for perennial crop breeding are therefore required.

Isolation of candidate genes of agronomic interest and development of specific molecular markers for application in molecular genotyping and marker-assisted selection (MAS) allow for both accelerated conventional breeding and gene-transfer programmes as strategies for genetic improvement. Expressed sequence tags (ESTs) are 5′- or 3′-end single-pass-sequenced portions of randomly isolated cDNA clones, which as such represent part of the transcribed region of the genome in given conditions. As a rapid approach for gene discovery and analysis of gene expression and regulation, data can also be exploited for the development of functional genetic markers. For *Musa*, a total of only 15 464 ESTs in *M. acuminata* and 5289 in *M. balbisiana* are currently publically available in GenBank (accessed March 2012). These datasets have been generated from a number of cultivars, plant tissues (Roux *et al*. 2008), during abiotic stress responses (Santos *et al*. 2005) and post-harvest ripening (Manrique-Trujillo *et al*. 2007). Only limited analysis of gene expression in response to biotic stresses has been reported (e.g. Van den Berg *et al*. 2004; Portal *et al*. 2011).

Highly variable microsatellites or simple sequence repeats (SSRs) are abundant in eukaryotic genomes, and may occur in both coding and non-coding regions (e.g. Tamana and Khan 2005). Typically they are reproducible, somatically stable, highly polymorphic, co-dominant, multi-allelic markers, with application in population genetics, genetic mapping and molecular breeding. Locus-by-locus *de novo* development is costly and time consuming, in contrast to mining from EST sequence databases. As EST–SSR markers originate from transcribed genes, they offer potential for analysis of functional diversity in populations and application in MAS, through utilization of markers that either originate from a gene responsible for a desirable phenotypic trait, or that co-localize with a particular quantitative trait locus (QTL) (Varshney *et al*. 2005). Applications of SSR markers in *Musa* have focused on evolution and taxonomy (e.g. Lagoda *et al*. 1998), genotyping (e.g. Crouch *et al*. 2001; Creste *et al*. 2003; Christelová *et al*. 2011), and, more recently, linkage map saturation (e.g. Hippolyte *et al*. 2010). In comparison with other important crops, however, still relatively few SSR markers have been developed for *M. acuminata* and *M. balbisiana* material (e.g. Kaemmer *et al*. 1997; Lagoda *et al*. 1998; Crouch *et al*. 2001; Buhariwalla *et al*. 2005; Creste *et al*. 2006, Cheung and Town 2007; Miller *et al*. 2010). Considering that alleles can be monomorphic or even absent when applied across cultivars, the number of useful SSR loci available remains limited.

This work describes the generation of an EST resource for *M. acuminata* and its mining for gene-derived SSR markers. The annotated ESTs were generated from two cDNA libraries constructed from BLSD-resistant *M. acuminata* ssp. *burmannicoides* var. Calcutta 4 (MAC4) and BLSD-susceptible *M. acuminata* subgroup Cavendish cv. Grande Naine (MACV) leaves *in vitro* infected with *Mf*. The wild diploid cultivar Calcutta 4 is widely employed in breeding programmes as a source of resistance to fungal pathogens and nematodes. It has also been used as a model for comparative genomics (Cheung and Town 2007; Lescot *et al*. 2008), functional genomics (e.g. Santos *et al*. 2005) and candidate resistance gene discovery (e.g. Azhar and Heslop-Harrison 2008; Miller *et al*. 2008). A subset of the EST–SSR marker loci was screened for polymorphism across *M. acuminata* accessions contrasting in resistance to *Mycosphaerella* leaf spot diseases.

## Materials and methods

### Bioassays

*In vitro*-derived, 6-month-old whole plants of *M. acuminata* Calcutta 4 (BLSD resistant) and Cavendish Grande Naine (BLSD susceptible) (*Musa* International Transit Centre accessions ITC0249 and ITC0654, respectively) were maintained in a greenhouse under a 12-h light/12-h dark photoperiod at 25 °C and 85 % relative humidity. Leaf disc materials (squares of 36 cm^2^) for the two contrasting *M. acuminata* cultivars were collected from the two youngest leaves and spray inoculated on the adaxial surface using conidiospore suspensions (3 × 10^3^ mL^−1^) of the *Mf* strain CIRAD89. Inoculated leaf discs were incubated in a climatic chamber at 25 °C, again under a 12-h light/12-h dark photoperiod. Calcutta 4 was shown to be highly resistant, with a typical incompatible response, whereas Cavendish was found to be highly susceptible, displaying symptoms of a compatible interaction. Seven replicate leaf discs were prepared to ensure sufficient material for RNA purification and microscopic examination following infection. The *in vitro*-infected leaf disc tissues were maintained for extended periods in a green, non-senescent state, according to Abadie *et al*. (2008).

### cDNA library construction

Two cDNA libraries were constructed, the first from a pool of RNA samples isolated from infected leaf discs at early time points in the incompatible interaction [4, 6, 7, 10, 12, 14 days after inoculation (DAI)] [*M. acuminata* ssp. *burmaniccoides* Calcutta 4 (MAC4)] and the second from pooled late time points in the compatible interaction (19, 25, 31, 39 DAI) [*M. acuminata* cv. Cavendish Grande Naine (MACV)]. This approach was adopted not only to generate EST resources, but also to potentially enrich the unigene set for genes involved in defence responses during this host–pathogen interaction. Collected leaf material was flash frozen in liquid nitrogen to prevent RNA degradation and stored at −80 °C. Total RNA was extracted from leaf tissue using the Trizol kit (Invitrogen, Carlsbad, CA, USA), according to the manufacturer's instructions. Total RNA quantification and quality analyses were conducted on an Agilent 2100 Bioanalyzer (Agilent Technologies, Palo Alto, CA, USA). Poly A^+^ RNA was isolated from total RNA using a MicroPoly(A)Purist™ mRNA Isolation Kit (Ambion, Austin, TX, USA), according to the manufacturer's instructions. Full-length cDNA libraries were constructed using the Creator SMART cDNA Library Construction kit (Clontech, Palo Alto, CA, USA). Poly A^+^ RNA quality was compared with an in-house control, and cDNA synthesized by reverse transcriptase, via long-distance polymerase chain reaction (PCR). High-quality cDNA was isolated via fractioning, digested with SfI and ligated to the plasmid cloning vector pDRN-LIB (Clontech). Transformation into *Escherichia coli* and recombinant selection on selection medium followed the manufacturer's protocols. Library qualities were examined by colony PCR and PCR amplification of plasmid inserts from randomly selected cDNA clones, with over 90 % showing inserts >400 bp. A total of 27 648 clones were prepared for each cDNA library and preserved as glycerol cultures.

### Sequence analysis

Randomly selected clones from each cDNA library were 5′-end single-pass di-deoxy-based Sanger sequenced in Brazil at the Universidade Católica de Brasília, Embrapa Recursos Genéticos e Biotecnologia and in Japan at the National Institute of Agrobiological Resources using BigDye chemistry (Applied Biosystems, Foster City, CA, USA). A total of 14 272 sequences were generated from the MAC4 library and 7623 from the MACV library. Sequence analysis began with base calling and quality assignment using the program Phred and a *Q* < 16 quality score (*Q*) threshold (Ewing and Green 1998). Low-quality sequences were removed using the program Lucy (Chou and Holmes 2001) and vectors were masked using Cross_Match (Ewing and Green 1998). Sequences were screened for contaminant *E. coli*, chloroplast and mitochondrial DNAs utilizing the SSAHA package (Website 1, **http://www.sanger.ac.uk/Software/analysis/SSAHA/**). The processed sequences were assembled into sequence consensi with the program TGICL (Pertea *et al*. 2003).

To annotate unique transcripts (unigenes) and identify putative functions, similarity searches were performed on assembled sequences using the Basic Local Alignment Search Tool (BLAST) suite of programs, version 2.2.24+ (Altschul *et al*. 1997), against distinct databases to identify protein functional categories [NCBI non-redundant sequence database (Website 2, **http://www.ncbi.nlm.nih.gov/COG/**); The Swiss-Prot Database (Website 3, **http://www.uniprot.org/downloads**, uniprot_sprot_ release of 2010 04 23); The TAIR Database: The Arabidopsis Information Resource (Website 4, **http://www.arabidopsis.org/**, Tair_9_pep_ release 2009 06 19); KOG (clusters of eukaryotic orthologous proteins from complete eukaryotic genomes); LSE (lineage specific expansions); and TWOG (clusters for two species)]. BLASTX criteria accounting for identity significance were that the alignment length should be >100 amino acids and the expected value (*E*) ≤ 1*E*^−10^. Species distribution for *Musa* unigenes was calculated via homology searches against all plant proteins in the NCBI NR database, based upon best hit for each analysed sequence. An *E*-value ≤ 1*E*^−3^ was set as the threshold to consider a BLAST hit significant. Unigene annotation based on protein domain comparisons with InterPro, Pfam and COG databases was conducted using InterProScan (version 4.5, **ftp://ftp.ebi.ac.uk/pub/software/unix/iprscan/**), HMMER3 (**http://hmmer.janelia.org**) and BLAST analyses. Gene placement prediction was performed using Metabolic pathway annotation against the Kyoto Encyclopedia of Genes and Genomes (KEGG) database (Kanehisa *et al*. 2004). Functional classification of annotated unigenes according to the categories of molecular function, biological process and cellular component was conducted using Blast2GO (Conesa and Götz 2008), following the gene ontology (GO) scheme (Consortium 2008).

Transposable elements (TEs) were identified during EST pre-processing steps using RepeatMasker Open-3.0 (**http://www.repeatmasker.org**) with the MIPS Repeat Element Database (Spannagl *et al.* 2007). Repeats were classified into superfamily, family and class according to version 4.3 of mips-REdat.

### Candidate gene expression at different time points

The isolated RNA samples used for cDNA library construction were normalized and 10 µg of each size separated via agarose gel electrophoresis (1.2 %) under denaturing conditions. Northern blot analyses of candidate gene expression at different time points during *Musa*–*Mf* interactions in the contrasting cultivars were carried out using Nylon Hybond N+ membranes according to the manufacturer's instructions. Polymerase chain reaction fragments of three selected cDNA clones of interest (GenBank accession numbers JK533438, JK545622 and JK535529) were labelled with α-^32^P dCTP via random hexanucleotide-primed DNA synthesis using the Megaprime^TM^ DNA Labelling System RPN 1607 (Amersham Biosciences, Piscataway, NJ, USA). Membrane hybridization signals were observed after exposure on an autoradiography Storm 820 imaging system (Amersham Biosciences, Piscataway, NJ, USA).

### *In silico* SSR identification and marker development

A computational search using the program Mreps (Website 5, **http://bioinfo.lifl.fr/mreps/**) was used to locate perfect SSRs across EST subsets (2186 ESTs from the MAC4 library and 2363 from the MACV library). Microsatellite detection required the presence of at least two repeating units (e.g. GC) spanning >10 bp. Flanking forward and reverse primers were designed using the program Primer 3 (Rozen and Skaletsky 2000).

In order to assess amplification and allele length polymorphisms, markers were evaluated using 20 diploid (AA) *M. acuminata* accessions belonging to the Embrapa Cassava and Fruits breeding programme collection, contrasting in resistance to Sigatoka diseases, and potential parentals for genetic map construction (Table 1). Genomic DNA was extracted from leaves of each accession using a modified mixed alkyl trimethyl ammonium bromide procedure (Gawel and Jarret 1991). Polymerase chain reactions were carried out in 13-µL volumes, using 3 ng of genomic DNA, 2.5 mM MgCl_2_, 0.2 mM dNTP mix, 0.5 µM primer, 1.25 U of *Taq* polymerase (Invitrogen) and 1× buffer. Polymerase chain reaction amplification was conducted with the following temperature cycling: denaturation at 94 °C for 5 min; 29 cycles of denaturation at 94 °C for 1 min, specific primer annealing temperature for 1 min and product extension at 72 °C for 1 min; plus a final elongation period of 7 min at 72 °C. Polymerase chain reaction products were initially checked for amplicon size and PCR specificity on 3.5 % agarose gels in 1× TBE buffer. Allele sizes were determined for products run against 10-bp molecular size markers (Invitrogen) on denaturing 6 % polyacrylamide gels using 7 M urea. Polymerase chain reaction products were visualized by silver staining according to the standard protocols (Creste *et al.* 2001). Polymorphism per locus was calculated via the polymorphism information content (PIC) calculator (Website 6, **http://www.liv.ac.uk/~kempsj/pic.html**).

**Table 1:** **Diploid (AA)** *M. acuminata* **accessions contrasting in resistance to** *Mycosphaerella* **leaf spot diseases selected for use in SSR marker validation**.

*M. acuminata* accession	Resistance/susceptibility to BLSD	Resistance/susceptibility to yellow Sigatoka
Calcutta 4	Resistant	Resistant
Lidi	Resistant	Resistant
0323-03	Resistant	Resistant
SH32-63	Resistant	Susceptible
1304-06	Resistant	Resistant
0116-01	Resistant	Resistant
Burmanica	Resistant	Resistant
Microcarpa	Resistant	Resistant
1741-01	Nd^a^	Resistant
9179-03	Nd	Resistant
1318-01	Nd	Resistant
4279-06	Nd	Resistant
Pisang Berlin	Partially resistant	Susceptible
Niyarma Yik	Susceptible	Susceptible
Raja Uter	Nd	Susceptible
Tjau Lagada	Susceptible	Susceptible
F2P2	Nd	Susceptible
Khai Nai On	Susceptible	Susceptible
Sowmuk	Resistant	Susceptible
Jaribuaya	Resistant	Susceptible

^a^Not defined.

## Results

### Bioassay

**Fig. 1:**
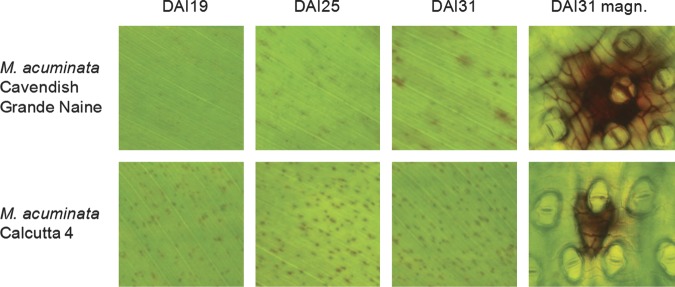
**Macroscopic and microscopic observation of infected tissues during the time course interaction with *Mf*.** Symptoms of apoptosis were apparent only in Calcutta 4 (19–31 DAI), while cell death attributed to necrotic disease stages was restricted to Cavendish Grande Naine (DAI31). DAI, days after inoculation; magn, magnified.

A highly reproducible *in vitro* infection procedure was developed to assess the level of resistance to *Mf* in *M. acuminata*. Two *Musa* genotypes were selected for their contrasting resistance responses to the fungal pathogen, with Fig. 1 showing significant phenotypic differences at the macroscopic level. Following inoculation with *Mf* conidiospore suspensions, early cellular responses (19 DAI) were observed in Calcutta 4, leading to the activation of apoptotic events that blocked fungal growth after ingression via the stomata. Apoptosis was limited to sub-stomatal cells, with no further cell death progression observed between 19 and 31 DAI. These observations are indicative of a complete arrest of fungal growth in Calcutta 4. In this early biotrophic infection phase, such rapid induction of sub-stomatal cell death would deprive the fungus of nutrients required for survival. By contrast, the infection time course in leaves of the genotype Cavendish Grande Naine revealed fungal penetration of the host, with infection of sub-stomatal cells advancing in the mesophyll, resulting in extensive cell death during later necrotrophic stages (Fig. 1, magnified image, DAI31).

### Unigenes

For the development of an EST dataset for *M. acuminata*, two full-length cDNA libraries were constructed, from MAC4 and MACV leaf tissue samples, both *in vitro* infected with *Mf*. The estimation of insert size via both restriction digestion with SfI and PCR amplification revealed averages in excess of 400 bp, showing that both cDNA libraries were of high quality.

From a total of 10 995 single-pass 5′-sequenced clones in the MAC4 cDNA library, vector trimming and quality analyses resulted in 9333 high-quality reads. In the case of the MACV cDNA library, from an initial 4157 clones, a total of 3962 high-quality reads were generated. Size distribution analysis revealed a mean length of ESTs following quality filtering and vector trimming of 370 bp for MAC4-derived ESTs and 494 bp for MACV-derived ESTs. The most common length distribution categories were between 201 and 500 bp for MAC4 ESTs, and between 401 and 500 bp in the case of MACV ESTs. All high-quality sequences were deposited in NCBI with GenBank accession numbers JK531581–JK540913 (MAC4) and JK542313–JK546274 (MACV).

**Fig. 2:**
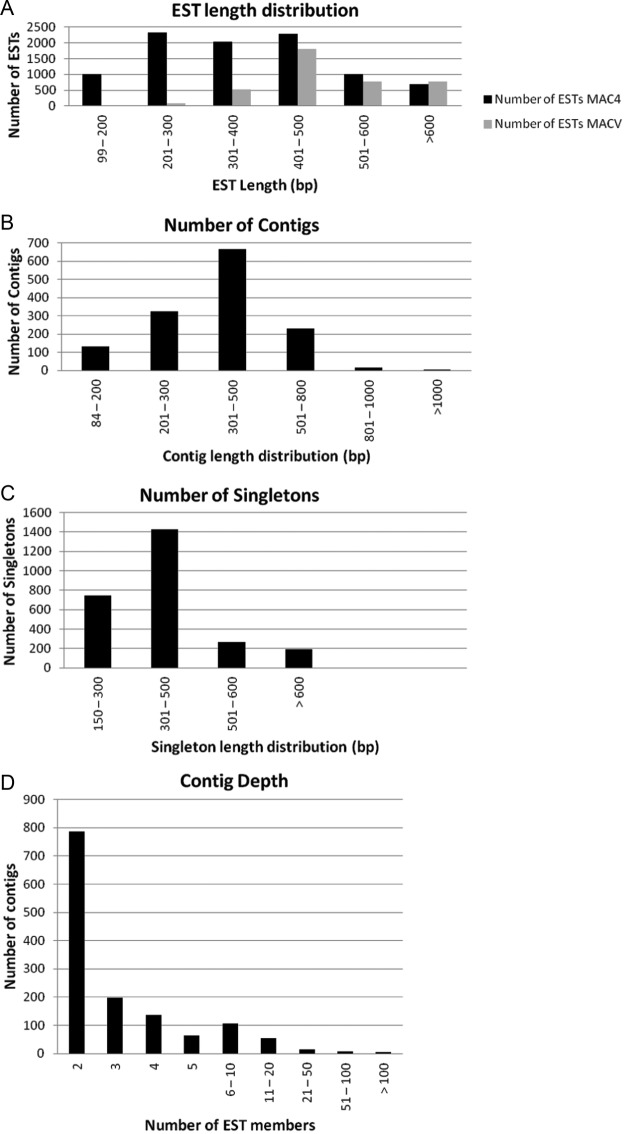
**Summary of EST quality and sequence assembly from combined MAC4 and MACV datasets.** (A) Length distribution of the *M. acuminata* ESTs; (B) length distribution of the assembled *M. acuminata* unigene contigs; (C) length distribution of the *M. acuminata* unigene singletons; (D) frequency and distribution of ESTs in assembled *M. acuminata* unigene contigs.

Assembly of high-quality *M. acuminata* ESTs from the two libraries generated 3995 non-redundant unigene clusters, consisting of 1368 contigs and 2627 singletons (1908 from MAC4 and 719 from MACV). Clustering resulted in an average of 16 EST sequences. As expected, contigs with fewer EST members were more represented than those composed of more ESTs (Fig. 2).

### Functional annotation and classification

**Fig. 3:**
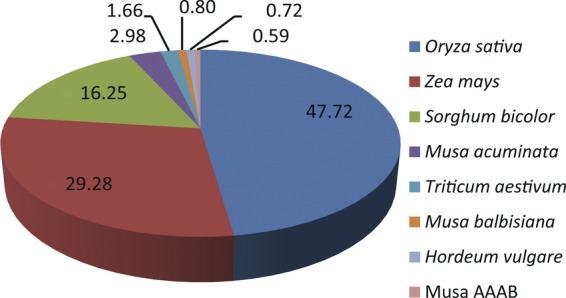
**Species distribution of *M. acuminata* unigenes shown as the percentage of the total homologous monocotyledon plant sequences.** The best BLAST hits of each sequence were analysed.

**Fig. 4:**
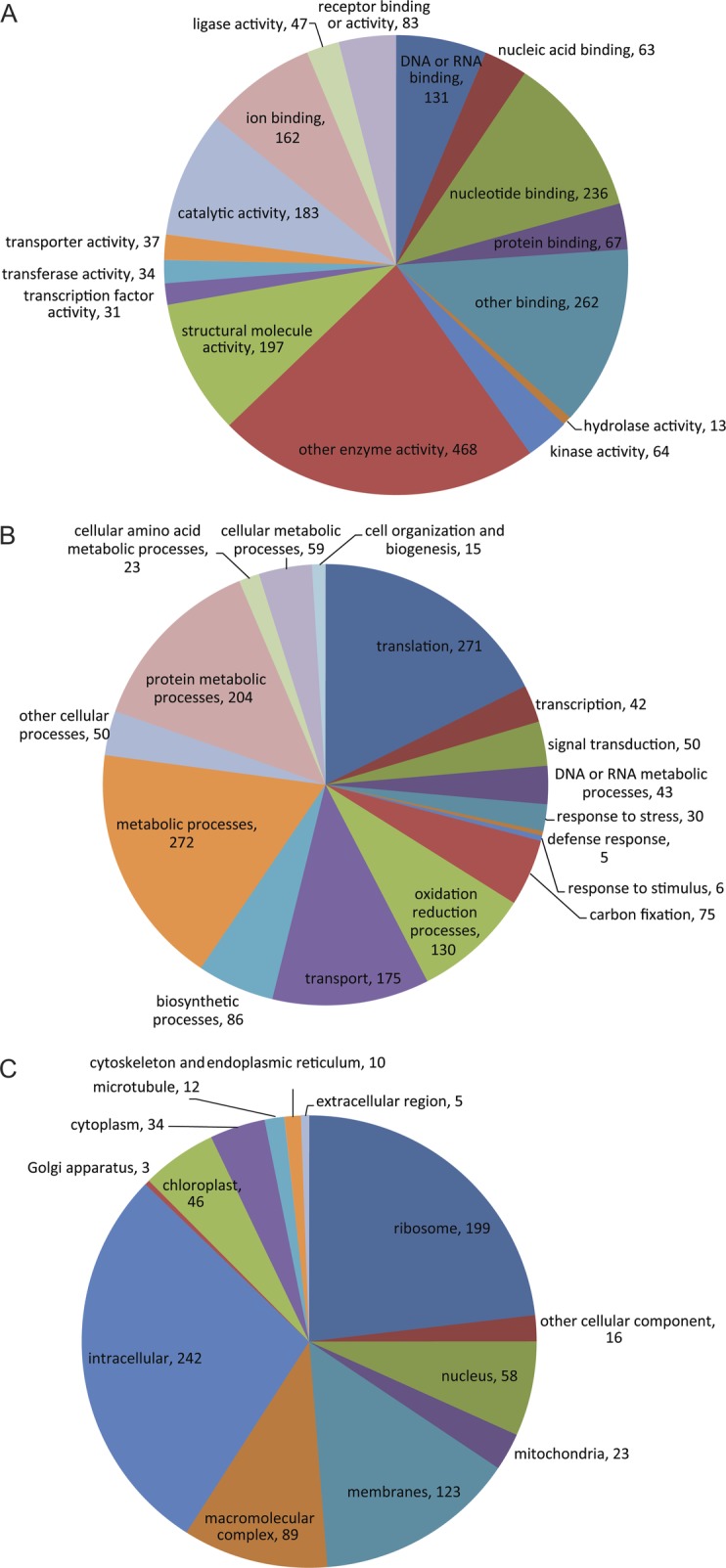
**Representation of *M. acuminata* unigenes classified in functional groups.** The GO hits were assigned to categories (A) molecular function, (B) biological process and (C) cellular component.

Expressed sequence tag annotation was conducted via the BLASTX algorithm-based alignment against the NCBI non-redundant sequence database, SwissProt, MIPS-*Arabidopsis*, GO and KOG. Conserved protein domains were also identified using InterproScan. A total of 2592 unigene sequences displayed significant homology to genes encoding proteins with known or putative function, 266 to genes encoding proteins with unknown function, and 1137 showed no significant homology to any sequences in the database. A total of 486 (12 %) matched genes in rice (*Oryza sativa*), 182 (5 %) matched genes in maize (*Zea mays*) and 247 (6 %) matched genes in sorghum (*Sorghum bicolor*) (Fig. 3). Only 4.1 % of BLAST hits (165 unigenes) originated from *Musa* NR database proteins, indicating considerable gene discovery for the genus. Gene ontology is employed to provide an organized vocabulary for describing unigenes according to categories (Ashburner *et al*. 2000). Functional annotation of the 3995 unigenes with InterproScan analysis identified a total of 543 GO terms. Unigenes were annotated with GO identifier into three principal categories: molecular functions (46.43 %), cellular components (19.21 %) and biological processes (34.34 %). Two unigenes (0.04 %) remained unclassified, possibly reflecting limited sequence length or that they are novel proteins. Details of assigned high-level GO terms are shown in Fig. 4. As unigenes could occasionally be assigned to more than one category, the combined total number of assigned GO mappings exceeded the number of unigenes analysed. In the molecular function category, the four most represented unigene functional classes were: other enzyme activity (468), other binding (262), nucleotide binding (236) and structural molecule activity (197). The principal functional classes observed in the biological function category belonged to metabolic process (272), translation (271), protein metabolic process (204) and transport (175). In the cellular component category, most unigenes coded for intracellular cell part (242), ribosome (199), membranes (123) and macromolecular complex (89).

#### KOG categories

A total of 2300 unigenes were assigned to KOG categories: amino acid transport and metabolism (72/1.8 %), carbohydrate transport and metabolism (110/2.8 %), cell cycle control, cell division, chromosome partitioning (28/0.7 %), cell motility, lipid transport and metabolism (1/0.02 %), cell wall/membrane/envelope biogenesis (28/0.7 %), chromatin structure and dynamics (23/0.5 %), coenzyme transport and metabolism (19/0.4 %), cytoskeleton (49/1.2 %), defence mechanisms (26/0.6 %), energy production and conversion (127/3 %), extracellular structures (8/0.2 %), function unknown (160/4 %), general function prediction (319/7.9 %), inorganic ion transport and metabolism (67/1.6 %), intracellular trafficking, secretion and vesicular transport (119/2.9 %), lipid transport and metabolism (56/1.4 %), nucleotide transport and metabolism (21/0.5 %), post-translational modification, protein turnover, chaperones (320/8 %), replication, recombination and repair (15/0.3 %), RNA processing and modification (89/2.2 %), secondary metabolite biosynthesis, transport and catabolism (53/1.3 %), signal transduction mechanisms (172/4.3 %), transcription (109/2.7 %), translation, ribosomal structure and biogenesis (289/7 %), and no hits (1696/42.5 %) [see ADDITIONAL INFORMATION 1 and 2].

#### Defence

Defence-related transcript functions identified according to the KOG classification across the unigenes comprised nine germin/oxalate oxidases (OXOs), three regulators of pathogen resistance responses of RPS2 and RPM1 genes, two dual-specificity phosphatases, two flavonol reductase/cinnamoyl-CoA reductases, two HVA22/DP1 gene product-related proteins, two macrophage migration inhibitory factors, a drought-induced protein, a bax-mediated apoptosis inhibitor TEGT/BI-1, a BPI/LBP/CETP family protein, a mercaptopyruvate sulfurtransferase/thiosulfate sulfurtransferase, a predicted protein tyrosine phosphatase, and a protein involved in the control of unknown local host defence mechanisms to pathogens.

#### Signal transduction

From a total of 172 predicted unigenes classified to the KOG category of signal transduction, a number are typically associated with plant immunity mechanisms. These included 20 unigenes characterized as ‘Receptor protein kinase containing LRR repeats’. Other significant findings were 20 serine/threonine protein kinases, two mitogen-activated protein kinase kinase (MAP2K) and five WRKY superfamily transcription factors. Further manual mining also revealed predicted unigenes typically associated with defence responses in plants: five isoflavone reductase/pinoresinol-lariciresinol reductase/phenylcoumaran benzylic ether reductases (phenylpropanoid/flavonoid pathway); 14 glutathione *S*-transferases (GSTs), two metallothioneins, three superoxide dismutases (SOD) (plant detoxification); one 1-aminocyclopropane-1-carboxylate synthase (ethylene biosynthesis); three β-1,3 glucanases (PR 2 proteins); one transcription factor containing NAC and TS-N domains (plant defence); one cysteine proteinase inhibitor B (cystatin B) (plant defence); and six glycolate oxidases [production of reactive oxygen species (ROS)].

#### Functional validation of defence-related gene expression

**Fig. 5:**
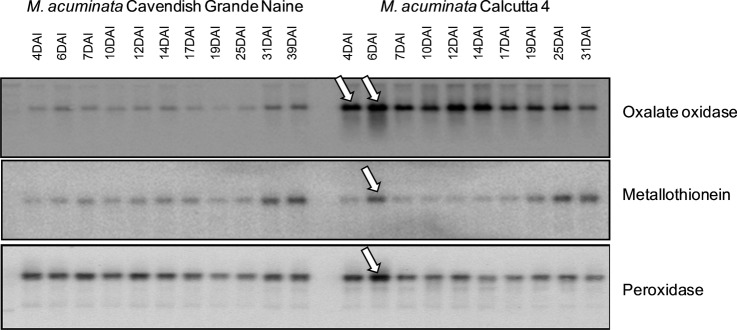
**Northern blot analysis of expression of selected defence-related *M. acuminata* unigenes during the time course interaction with *Mf*.** Arrows show differential gene expression between the tested cultivars for each gene probe.

By employing RNA samples used for cDNA library construction, a northern blot time course was conducted to assess differential induction of a set of identified defence-related genes following infection of each *M. acuminata* genotype. Selected candidate genes comprised an OXO (clone accession number JK533438), one representative of the metallothionein type 2 gene family (JK545622) and one peroxidase (JK535529). The time course for the analysis of gene expression during the interaction analysis covered early (4 DAI) until late time points (31 DAI in the Calcutta 4–*Mf* interaction, 39 DAI in the Cavendish Grande Naine–*Mf* interaction). Analysis revealed differences in the pattern of expression induction of the selected genes between the incompatible and compatible *Musa*–*Mf* interaction. Early induction of the OXO (4 DAI, 6 DAI), the metallothionein (6 DAI) and the peroxidase (6 DAI) in Calcutta 4 cells correlated with the observed apoptotic events (Fig. 5), suggesting their involvement in a rapid activation of defence responses. By contrast, no significant early induction of the three genes was observed in Cavendish Grande Naine, with an increased expression of both OXO and metallothionein only 31 and 39 DAI, and a relatively constant expression of peroxidase throughout the time course.

#### KEGG pathways

To identify biological pathways represented among the unigenes, enzyme commission numbers derived from BLASTX alignments were mapped against the KEGG database. A total of 312 unigenes were identified in the pathway maps, with the categories genetic information processing and metabolism accounting for 86 % of the unigenes (Table 2). The five most represented pathway subcategories were: translation; energy metabolism; folding, sorting and degradation; carbohydrate metabolism; and amino acid metabolism.

**Table 2:** **Summary of** *M. acuminata* **unigenes mapped in KEGG pathways**.

Pathway categories	Unigenes
Genetic information processing	143
Metabolism	128
Cellular processes	18
Organism systems	14
Environmental information processing	9
Unigene total	312

### Transposable elements

To analyse the abundance and diversity of expressed TEs, EST pre-processing employed RepeatMasker (version open-3.2.8), with classification to type level according to the database mips_REdat_4.3. Differences in the proportion of retrotransposons (85 %) and transposons (16 %) were observed. Class I TEs were classified into long terminal repeat (LTR), non-LTR and retrotransposon type, while Class II TEs were classified only to transposon type. Table 3 summarizes the number of EST sequences containing each TE type.

**Table 3:** **Abundance and diversity of expressed TEs in** *M. acuminata* **EST datasets**.

Class	Type	Number of ESTs
I	LTR	220
I	Non-LTR	21
I	Retrotransposon	16
II	Transposon	48
Total		305

### Genic-SSR marker development

Computational mining of *M. acuminata* ESTs (2186 from the MAC4 library and 2363 from the MACV library, with a total size of 2104 Mbp) identified SSRs across 13.7 % of sequences. For 303 out of 624 SSR-positive sequences, PCR primers could be successfully designed, for potential use as molecular markers based on repeat length polymorphisms (Table 4). A total of 12.5 % of analysed MAC4 ESTs contained SSRs, with five classes identified. The trinucleotide repeats appeared the most abundant (54.1 %), followed by di- (31.6 %), tetra- (6.7 %), hexa- (5.3 %) and penta-nucleotide repeats (2.3 %). The most abundant trinucleotide repeat motifs were GAA, CTC, AAG, AGA, CCT, CAG, GAG, GAT, CAC and AGG, accounting for 68 % of such repeats. Of the dinucleotide repeat motifs, GA, AG, TC and CT accounted for 78.5 % of repeats. Tetranucleotide repeats were less abundant, with the majority of motifs in equal abundance (11.1 % each), with the exception of the more frequent GAGG motif (22.2 %). Penta- and hexa-nucleotide repeats represented the least abundant in Calcutta 4, with equal abundance observed for each motif. Analysis of MACV ESTs revealed 14.8 % containing SSRs. In contrast to Calcutta 4, a greater array of repeat classes was observed, from di- through to hendeca-nucleotide repeats. As in the case of Calcutta 4, trinucleotide repeats were the most abundant (57.6 %). These were followed, in decreasing frequency, by di- (25.3 %), tetra- (7.1 %), hexa- (4.7 %), penta- (3.5 %), hepta- (0.6 %), octa- (0.6 %) and hendeca-nucleotide repeats (0.6 %). Trinucleotide repeat motifs included, in decreasing prevalence, CTC, AGA, TTC, AAG, GAA, CCT, GGA and TCT, representing 50.0 % of tri-repeats. The most common dinucleotide repeat motifs GA, TC, AG and CT, also common in Calcutta 4, accounted for 88.4 % of repeats. Tetranucleotide repeat motifs were all present in equal abundance (8.3 % each). Penta- and hexa-nucleotide repeat motif types were also each present in equal abundance per class, at 16.7 and 12.5 %, respectively. In the case of hepta-, octa- and hendeca-nucleotide classes, only one motif type per class was observed. In general, the shorter the nucleotide core sequence, the greater were the number of repeats observed. In the case of Calcutta 4 there were an average of 9.4 repeats for di-nucleotide motifs, 5.2 for tri-, 3.5 for tetra-, 3.3 for penta- and 3.4 for hexa-motifs. Similarly, for Cavendish Grande Naine there were an average of 9.6 repeats for di-, 5.4 for tri-, 3.9 for tetra-, 3.1 for penta-, 3.8 for hexa-, 3.1 for hepta-, 3 for octa- and 3.1 for hendeca-motifs.

**Table 4:** **Overview of SSR repeat abundance in** *M. acuminata* **ESTs and primer design statistics**.

	*M. acuminata* Calcutta 4	*M. acuminata* Cavendish Grande Naine
Sequences analysed	2186	2363
Sequences with SSR repeats	273	351
Bases analysed	1 021 899	1 082 286
Bases with repeats	5478	7171
Primer pairs designed	133	170
Failed primers	51	67

Of the 303 EST-derived SSR markers for which primers could be designed, 149 yielded reproducible PCR amplicons [see ADDITIONAL INFORMATION 3]. A total of 75 (24.7 %) were identified with consistent amplification and as polymorphic loci when tested, initially on agarose gels and subsequently on polyacrylamide gels, across the contrasting *M. acuminata* accessions (Table 5). A total of 289 alleles were scored across these polymorphic loci. Fourteen polymorphic loci possessed two alleles across the tested accessions; 21 loci showed three alleles; 17 loci showed four alleles; 13 loci showed five alleles; six loci showed six alleles; three loci showed seven alleles; and one locus displayed eight alleles. The PIC values ranged from 0.08 to 0.81, with an average value of 0.50.

**Table 5:** **Characteristics of polymorphic microsatellite loci isolated from** *M. acuminata* **Calcutta 4 and Cavendish Grande Naine EST data**.

Locus name	SSR repeat motif	SSR locus length (bp)	Obtained allele size range (bp)	Allele number	He	Ho	PIC value
MA41	AG	27	350–390	8	0.83	0.63	0.81
MA45	TCTT	16	110–120	3	0.6	0.88	0.53
MA47	CTC	16	140–160	2	0.48	0.81	0.37
MA412	GAA	25	350–360	3	0.67	0.57	0.59
MA413	CTC	16	240–250	3	0.55	0.42	0.48
MA418	GAG	13	320–330	4	0.7	0.61	0.64
MA425	AG	21	220–250	7	0.79	0.79	0.76
MA426	TAAT	14	150–160	3	0.27	0.22	0.25
MA428	AAG	36	200–220	4	0.64	0.33	0.57
MA432	CAC	13	300–320	5	0.67	0.76	0.61
MA435	GAA	19	330–350	4	0.71	0.83	0.66
MA440	TTC	19	170–175	2	0.19	0.13	0.17
MA441	CT	13	100–110	5	0.63	0.52	0.58
MA443	TTC	18	310–340	5	0.71	0.58	0.67
MA444	CT	33	260–280	3	0.29	0.35	0.26
MA446	TC	14	140–150	3	0.61	0.27	0.53
MA452	AGA	21	170–180	5	0.7	0.73	0.66
MA453	CGC	12	330–335	2	0.50	0.21	0.37
MA455	GAA	20	240–250	3	0.54	0.23	0.45
MA466	CAG	16	170–180	4	0.75	0.67	0.7
MA472	GAGAG	15	300	4	0.60	0.35	0.54
MA476	AC	13	200–210	4	0.72	0.58	0.67
MA479	CCT	13	340–350	4	0.3	0.38	0.26
MA489	AT	14	260–300	3	0.44	0.23	0.37
MA490	CAC	16	190–200	3	0.57	0.47	0.49
MA492	AT	23	175–180	5	0.69	0.75	0.65
MA493	CTC	12	310–320	2	0.5	0.5	0.37
MA495	GAA	16	180–220	2	0.42	0.00	0.33
MA4100	AG	14	320–335	3	0.38	0.48	0.33
MA4104	GA	47	300–320	4	0.61	0.38	0.55
MA4110	TCT	30	280–300	6	0.74	0.25	0.70
MA4111	AGC	26	375–290	4	0.73	0.52	0.68
MA4116	CT	23	380–410	5	0.72	0.65	0.68
MA4128	CTT	25	210–220	3	0.47	0.47	0.38
MACV11	CAG	20	210–220	4	0.5	0.5	0.46
MACV15	GA	22	270–290	6	0.81	0.33	0.79
MACV20	TC	12	340–360	5	0.72	0.83	0.66
MACV21	TCA	36	340–360	4	0.47	0.57	0.43
MACV27	TC	36	290–310	5	0.73	0.63	0.68
MACV29	TAAT	14	140–150	2	0.39	0.13	0.32
MACV36	CT	33	240–270	5	0.78	0.77	0.74
MACV37	AGC	21	100–110	3	0.1	0.1	0.09
MACV42	AGA	24	320–330	3	0.56	0.25	0.48
MACV47	GA	32	320–320	3	0.64	0.61	0.56
MACV49	AGGCGA	21	270–275	2	0.39	0.33	0.31
MACV50	TC	13	130–140	3	0.1	0.1	0.09
MACV51	CT	19	180–200	4	0.4	0.48	0.37
MACV54	CAG	12	160–180	4	0.68	0.7	0.62
MACV55	AGA	18	170–190	6	0.73	0.83	0.69
MACV62	TTTTTA	28	250–280	3	0.53	0.1	0.43
MACV63	TGC	25	120–140	6	0.72	0.67	0.67
MACV73	CTCTC	16	220–225	2	0.41	0.42	0.33
MACV77	GCC	12	90–115	4	0.58	0.54	0.52
MACV81	GAA	15	200–205	2	0.28	0.05	0.24
MACV87	TCC	15	110–130	4	0.69	0.83	0.64
MACV88	GAA	27	250–300	5	0.76	0.71	0.72
MACV96	TCT	15	180–210	5	0.75	0.57	0.70
MACV99	GA	13	240–260	2	0.08	0.08	0.08
MACV104	AG	14	290–330	7	0.81	0.65	0.79
MACV109	GGA	23	385–390	2	0.08	0.00	0.08
MACV111	CTACA	16	130–150	2	0.63	0.55	0.60
MACV112	CTC	12	330–360	3	0.78	0.74	0.74
MACV115	AAG	12	150–165	6	0.59	0.55	0.51
MACV128	ATGCTC	20	420–450	4	0.43	0.27	0.39
MACV132	CTC	31	280–290	4	0.58	0.65	0.53
MACV134	CCT	13	320–335	3	0.51	0.78	0.42
MACV139	GA	17	90–120	7	0.75	0.95	0.71
MACV148	CTC	19	150–170	5	0.69	0.57	0.63
MACV151	TTC	21	270–290	5	0.75	0.87	0.71
MACV154	AAG	12	130–150	3	0.12	0.13	0.12
MACV155	TC	21	330–350	3	0.63	0.26	0.56
MACV157	GGA	13	140–155	2	0.29	0.35	0.25
MACV161	GCA	12	110–120	3	0.61	0.42	0.53
MACV162	ATCTG	15	190–200	2	0.15	0.17	0.14
MACV169	TC	37	150–170	6	0.79	0.75	0.76

Polymorphism was evaluated across 20 *M. acuminata* accessions.

HE, expected heterozygosity under Hardy–Weinberg expectations; HO, observed heterozygosity; PIC, polymorphism information content.

## Discussion

The objectives of this work were to generate an EST resource for studying functional genes in *M. acuminata*, which also included transcripts expressed in banana–*Mf* interactions during compatible and incompatible reactions. We also pursued the development of gene-based microsatellite markers as a resource for genetic mapping, diversity characterization and MAS of specific traits in conventional breeding populations.

### Unigenes

In total, 9333 high-quality ESTs were generated from MAC4 and 3964 from MACV. At the time of analysis in December 2011, only 15 464 ESTs were publically available for *M. acuminata*. This study therefore contributes almost a two-fold increase in EST resources for this species. BLASTX homology searches of the 3995 *M. acuminata* unigenes against monocotyledonous plant proteins in the NCBI NR database revealed 28.4 % of unigenes as potentially novel and exclusive to *M. acuminata*, with only 4.1 % of BLAST hits to existing *Musa* NR database proteins. This dataset therefore provided a significant contribution of value for gene discovery and validation of function for the genus.

Functional categorization assigned a large number of unigenes to involvement in intracellular cell components, membranes, organelles, metabolic processes, translation, transport, oxidation and reduction processes, enzyme activity, binding, structural molecule activity and catalytic activity. Given the still limited characterization of gene expression during banana–*Mf* interactions (e.g. Portal *et al*. 2011), a strategy for potential enrichment of *Musa* EST resources to also include genes involved in defence responses was employed. Given that defence responses typically occur earlier in incompatible rather than compatible interactions, distinct time points for cDNA library preparation were chosen to reflect such expected differences. Although the sequences encoding activities related to response to stress, defence response and signal transduction were less represented, numerous unigene sequences potentially involved in plant effector-triggered immunity (ETI) and pathogen-associated molecular pattern (PAMP)-triggered immunity (PTI) were characterized. Pathogen-associated molecular pattern-triggered immunity is considered to be based upon interactions between host pattern recognition receptor-like kinases and conserved PAMPs (Nürnberger and Kemmerling 2009), conserved across a microbial class and essential in fitness. Pathogen-associated molecular pattern-triggered immunity involves activation of a mitogen-associated protein kinase cascade and WRKY transcription factors (TFs), conferring resistance to the majority of potential pathogens. Effector-triggered immunity (Jones and Dangl 2006) is based upon co-evolution of plant resistance R-protein receptors and specific pathogen effector molecules, conferring resistance at the intra-specific level. Many downstream signal transduction components are shared between PTI and ETI, including an oxidative burst via the production of ROS and changes in plant hormone levels. Mitogen-activated protein kinase signalling cascades also occur in both PTI and ETI, with variations in duration probably responsible for differential downstream responses in the two immunity branches (Tsuda and Katagiri 2010). Overall, a number of expressed genes potentially involved in different pathways in PTI or ETI were identified, the most abundant of which included host receptor genes involved in PAMP or pathogen effector recognition, unigenes involved in signalling mechanisms, phenylpropanoid/flavonoid pathway genes, phytohormone biosynthesis genes, pathogenesis-related protein coding unigenes and genes involved in plant detoxification.

#### Host receptor genes and signal transduction

Distinct plant *R*-gene families are recognized as involved in ETI, based upon protein domain structure and biochemical function. The most abundant class encodes cytoplasmic receptor proteins containing nucleotide binding site-leucine-rich repeat (NBS-LRR) domains (Hammond-Kosack and Jones 1997). In rice, ∼400 NBS-LRR genes have been characterized, with around 150 present in the *Arabidopsis* genome (McHale *et al.* 2006). Conservation of motifs within *R*-genes, such as those present within NBS-LRR domains, have facilitated amplification in diverse plant taxa. Such work has been reported in *Musa*, with large-scale analyses of NBS-LRR *R*-gene family RGA diversity across the genus (e.g. Miller *et al.* 2008; Mohamad and Heslop-Harrison 2008). In the current study, three regulators of pathogen resistance responses of NBS-LRR *R-*genes RPS2 and RPM1 genes were identified in the transcribed unigene dataset. The RPM1 protein is known to be associated with the host plasma membrane (Boyes *et al.* 1998), as is RPS2 (Mackey *et al.* 2003), where they recognize modification in the *Arabidopsis thaliana*negative regulator RPM1 interacting protein 4 (RIN4), target of *Pseudomonas syringae* type III bacterial effector proteins (Mackey *et al.* 2002), triggering the hypersensitive response (HR) or programmed cell death of infected cells, characterized by the appearance of small necrotic lesions at infection sites. Other known plant *R*-gene classes include extracellular LRRs anchored by transmembrane domains (receptor-like proteins), extracellular LRRs linked to cytoplasmic serine–threonine kinase domains, intracellular serine–threonine kinases and proteins with a coiled-coil domain anchored to the cell membrane. EuKaryotic orthologous group-based analysis predicted a total of 20 unigenes with function assigned as ‘Receptor protein kinase containing LRR repeats’. Over 600 such receptor-like kinases (RLKs) have been characterized in *Arabidopsis* (Shiu and Bleecker 2001), with the disease ETI resistance gene *Xa21* being one of the earliest examples from this class, conferring durable resistance to *Xanthomonas oryzae* pv. *oryzae* (Song *et al.* 1995). In a previous study in *M. acuminata* (Miller *et al.* 2011), sequence similarity analysis of amplification products generated using degenerate primers for RLKs identified numerous sequences with significant similarity to *R*-gene and RGA sequences for this class. A total of 20 serine/threonine protein kinases were also identified on the basis of KOG function assignment. Examples of such kinases include the intracellular cytoplasmic *R*-gene *Pto*, which was the first *R*-gene in tomato (*Solanum lycopersicum*) proved to confer resistance to *Pseudomonas syringae* pv. *tomato* strains that express the *AvrPto* gene (Martin *et al.* 1993). Defence reactions associated with HR and programmed cell death are considered to be induced following *AvrPto* recognition in the presence of an NBS-LRR protein known as *Prf*, which is present in the *Pto* kinase gene cluster. Other significant findings in relation to unigenes typically involved in signal transduction from pathogen recognition to defence gene expression included two MAP2K and five WRKY superfamily transcription factors.

#### Phenylpropanoid/flavonoid pathway

Phenylpropanoids in plants are involved in a number of defence responses, acting as antimicrobial compounds (phytoanticipins and phytoalexins) and molecules involved in signalling (Dixon *et al*. 2002; Naoumkina *et al.* 2010). EuKaryotic orthologous group classification revealed five isoflavone reductase/pinoresinol-lariciresinol reductase/phenylcoumaran benzylic ether reductases. Isoflavone reductase is an enzyme required for biosynthesis of the phytoalexin pterocarpan. Monolignols serve as precursors of plant lignins and lignans, which are composed of phenolic compounds and are involved in physical and chemical plant defence mechanisms. Cinnamoyl-CoA reductase is the first enzyme specific for monolignol synthesis. EuKaryotic orthologous group data identified two unigenes encoding this enzyme.

#### Pathogenesis-related proteins

Pathogenesis-related (PR) proteins were initially observed in tobacco (*Nicotiana tabacum*) and are now known to accumulate in diverse plant hosts when under pathogen attack. These structurally and functionally diverse proteins have been classified into 17 families (van Loon *et al*. 2006). Given that both HR observed in incompatible plant–pathogen interactions and subsequent systemic acquired resistance (SAR) to diverse pathogens are associated with accumulation of PR proteins in local and systemic tissues, such proteins are believed to contribute to resistance. Our unigene set included three β-1,3 glucanases, which are recognized as PR-2 family members. This widely studied family has been reported to limit activity in diverse fungal pathogens, through degradation of the cell wall component β-1,3 glucan. Up-regulation of β-1,3 glucanases in incompatible interactions has been reported (Elvira *et al*. 2008), and over-expression analyses have confirmed involvement in resistance. For example, PR2 genes from soybean (*Glycine max*) have been shown to confer resistance in potato (*Solanum tuberosum*) to *Phytophthora infestans* (Borkowska *et al*. 1998) and in kiwi (*Actinidea deliciosa*) to *Botrytis cinerea* (Grover and Gowthaman 2003). Similarly, a PR2 gene from potato increased resistance to both *Fusarium oxysporum* and *Fusarium culmorum* in flax (*Linum usitatissimum*) (Wróbel-Kwiatkowska *et al*. 2004).

#### Germin OXOs

Numerous germin OXOs were encountered in the unigene sets. Within the germin protein family, OXOs have been reported to play roles in calcium regulation, oxalate metabolism and response to pathogenesis (Davidson *et al*. 2009). Evidence for the latter includes up-regulation in cereals in response to powdery mildew (Zhou *et al*. 1998) and co-segregation of markers for OXO genes and with rice blast resistance QTLs (Wu *et al*. 2004). Oxalate oxidases can catalyse the conversion of ROS to H_2_O_2_ (Requena and Bornemann 1999), important components of HR in plants. Hydrogen peroxide is involved in cell wall cross-linking and messenger activity for activation of defence genes, triggering SAR. Also reported as a molecule necessary for phytoalexin biosynthesis, H_2_O_2_ has been shown to have direct antimicrobial activity, causing oxidation of invading pathogens (Wei *et al*. 1998). Our northern blot data revealed early increased expression (4–6 DAI) of OXO in resistant Calcutta 4 only, suggesting a possible involvement in ROS and associated HR components.

#### Plant detoxification

EuKaryotic orthologous group-derived mining revealed 14 GSTs in the unigene sets. Glutathione *S*-transferases appear to be ubiquitous in plants, with a function in endogenous and xenobiotic compound detoxification, such as herbicides (Hayes and McLellan 1999). Up-regulation has been shown in individual GSTs during pathogen attack in numerous plant species (e.g. Mauch and Dudler 1993; Alvarez *et al.* 1998), with likely involvement in detoxification of products of oxidative stress during HR, thus limiting both cell damage and the extent of cell death. Expression in *Mf–M. acuminata* compatible interactions has recently been reported (Portal *et al*. 2011). The potential role of GSTs in cell signalling pathways has also been suggested, with a GST from parsley involved in UV-dependent signal transduction (Loyall *et al.* 2000).

Metallothioneins are low-molecular-weight polypeptides rich in cysteine residues. Present across prokaryotes and eukaryotes, they play a role in detoxification and homeostasis, sequestering metal ions such as Cu^2^, Zn^2^ and Cd^2^, and preventing mutations (Hamer 1986; Robinson *et al*. 1993). Up-regulation has been observed in plants in response to increased metal concentrations (Hsieh *et al*. 1995). Since plants experience oxidative stresses following pathogen infection, it has also been argued that this protein family might be associated with regulation of intracellular redox potential and oxygen detoxification (Hamer 1986; Choi *et al*. 1996), protecting cells from damaging effects of ROS. Previous reports also indicate differential regulation of metallothioneins after viral infection in tobacco (Choi *et al*. 1996), temperature stress (Hsieh *et al*. 1995) and foliar senescence. Four distinct types (MT1–MT4) have been described in plants, according to distribution of cysteine residues (Robinson *et al*. 1993). Expression of MT1 is generally more associated with vascular tissues and roots, MT2 with shoots and leaves, MT3 with leaves and mature fruits, and MT4 with seed tissues. Liu *et al*. (2002) reported isolation of MT2 and MT3 in banana, with expression influenced in response to ethylene and metals. More recent examination of transcripts has reported that metallothionein-like genes are abundant in *M. acuminata* Calcutta 4 (Santos *et al*. 2005). Our study confirmed this, with isolation of MT2 and MT3 unigene sequences derived from contigs with considerable numbers of EST members. Northern blot data showing early expression of type 2 metallothionein-like proteins only in Calcutta 4 suggest involvement in cell protection ROS-scavenging during HR responses. By contrast, it is possible that late expression in Cavendish Grande Naine may indirectly reflect increased ROS during the fungal necrotrophic disease phase. Necrotrophs have been reported to induce ROS accumulation in plant hosts as a mechanism for promoting pathogen access to nutrients through triggering host programmed cell death (Govrin and Levine 2000).

#### Peroxidases

A number of peroxidases were also observed in the unigene sets. In addition to auxin metabolism, cell wall reinforcement and phytoalexin synthesis, such enzymes are also typically involved in ROS metabolism during defence responses (Almagro *et al*. 2009). Recent histochemical analysis of peroxidase and H_2_O_2_ accumulation during *Mf*–*M. acuminata* interactions reported a peaked accumulation of both in resistant *M. acuminata* Calcutta 4 at 10 days after conidial inoculation of detached leaf material, with no accumulation in susceptible Cavendish Grande Naine and partially susceptible Pisang Madu (Cavalcante *et al*. 2011). The marked early peroxidase gene expression induction 6 DAI observed only in resistant Calcutta 4 is in agreement with these previous findings, and again correlates with HR-like responses observed in this genotype.

### Transposable elements

Transposable elements are known to occur in all living organisms, and can occupy over 50 % of nuclear DNA. Given that these elements display mobility, they are important in plant evolution, through creation of novel genes or modifying gene function (Bennetzen 2000). In the case of vegetatively propagated crops such as banana, it is therefore likely that some somaclonal variation events can be due to such TE activity. Classification of eukaryotic TEs is based on the mode of transposition, with RNA-mediated TEs (Class I) and DNA TEs (Class II). Class I TEs can be divided into subclasses: long terminal repeats (LTRs), retroelements without LTRs (the long interspersed nuclear elements (LINEs) and the small interspersed nuclear elements (SINEs)) and TRIMs (Terminal-repeat Retrotransposons In Miniature). Class II TEs include the MITEs (Miniature Inverted-repeat Transposable Elements) (Feschotte *et al.* 2002). Our results revealed a predominance of retrotransposons to transposons. Similar distributions of DNA repeats have recently been reported in *M. acuminata* Calcutta 4, based on low-depth 454 sequencing of genomic DNA (Hribová *et al.* 2010).

### Markers

The development of genomic libraries enriched for SSRs is typically expensive and labour intensive, in contrast to data mining in ESTs. Expressed sequence tag-derived SSR markers enable enrichment of genetic maps with gene-based markers (Kota *et al.* 2001), as opposed to anonymous genomic DNA-derived SSRs which are predominantly derived from intergenic regions. Given that markers are isolated from coding regions, conservation is expected to be high, such that these EST–SSR markers are generally also transferable to related species (e.g. Gupta *et al*. 2003). The gene-based marker tools developed in this study for *Musa* also serve as a resource for diversity characterization and downstream marker-assisted breeding using markers for traits. Work is ongoing in the research community for the development of suitable segregant populations for traits of interest (Amorim *et al.* 2009; Dochez *et al.* 2009; Lorenzen *et al.* 2011). Linkage disequilibrium mapping is a potential alternative route for identifying genes for traits of interest in *Musa* (Heslop-Harrison and Schwarzacher 2007), which, while not dependent upon crosses and progeny maintenance, requires hundreds of plant accessions and thousands of genetic markers. The SSR markers designed in our work are also applicable for such a study. In general, the frequency and distribution of SSRs in ESTs and in genomic sequences differ, with dinucleotides typically more abundant in genomic survey sequences and trinucleotides more common in ESTs (e.g. La Rota *et al*. 2005; Varshney *et al.* 2005; Miller *et al.* 2010). In our study, tri-nucleotide repeat motifs (an average of 55.8 % across both EST datasets) were indeed more abundant than di-nucleotide motifs (average of 28.4 %). All other motifs, from tetra- to hendeca-repeats, were only poorly represented. Such a predominance of trinucleotides probably reflects the fact that such motifs in gene regions will avoid frameshift mutations which would cause changes at the protein level. Simple sequence repeat mining criteria in software may also distort real differences in motif abundance (Varshney *et al*. 2005). A total of 75 out of 303 tested SSR marker primer pairs were reproducibly polymorphic when tested across *M. acuminata* accessions contrasting in resistance to Sigatoka diseases, complementing the previous work by our group (Miller *et al.* 2010). Similar polymorphism rates have been observed in other crop species such as wheat and cotton (Eujayl *et al*. 2002; Han *et al*. 2006). Polymerase chain reaction amplification failed, however, for 106 primer pairs. Possible reasons include SSR extension across splice sites, poor sequence quality or chimeric DNA (Varshney *et al.* 2005). It has been reported that EST-derived SSR markers show less polymorphism than genomic sequence-derived SSRs, as a result of conservation in gene regions (Raju *et al.* 2010). Indeed, from a total of 75 loci, only 289 alleles were observed, with an average of 3.8 alleles per locus and an average PIC of 0.5. Considering that a total of 303 potentially functional SSR markers were identified from a subset of 4549 ESTs in the present study, it is possible to estimate approximately a further 1000 markers that could be derived from the 15 464 publically available *M. acuminata* ESTs. Given the advent of next-generation sequencing-derived gene expression sequence data, however, this number looks set to increase considerably.

## Conclusions and forward look

This study contributes considerably to publically available EST resources for *M. acuminata*, providing a unigene set of 3995 sequences derived from accessions Calcutta 4 and Cavendish Grande Naine during incompatible and compatible interactions with *Mf*. Genes was characterized according to the KOG-based classification, Interpro-based domain identification and GO category assignment. A large set of genic-SSR markers was developed, with polymorphic markers applicable for genetic map enrichment, diversity characterization and downstream marker-assisted breeding. In summary, it is anticipated that this dataset contributes to genomic resources for *Musa*, with downstream application in genetic improvement. Ongoing next-generation sequencing-based investigation of gene expression (including transcription profiling) in *Musa*–pathogen interactions by our group will offer potential for further elucidation of gene expression during plant immune responses, and will contribute to validating annotated gene models in the *Musa* whole-genome sequencing project.

## Additional information

The following additional information is available in the online version of this article –

File 1: *Musa acuminata* unigene assignment to KOG categories.

File 2: EuKaryotic orthologous group category abundance of *M. acuminata* unigenes.

File 3: Details of 303 *M. acuminata* genic-SSR markers, validated for polymorphism across 20 diploid accessions.

## Accession numbers

High-quality 5′ single-pass ESTs for 9333 cDNA clones from the MAC4 library and 3962 from the MACV library have been deposited in the GenBank database (**http://www.ncbi.nlm.nih.gov/dbEST/**) [accession numbers: JK531581–JK540913 (MAC4); JK542313–JK546274 (MACV)].

## Sources of funding

This work was funded by the IAEA, Austria (Project 13187), FINEP, Brazil (Project 0107060900/0842/07), CNPq, Brazil (Projects 680.398/01-5 and 506165/2004-3). M.A.N.P. was supported by a fellowship from the CNPq.

## Contributions by the authors

M.A.N.P. participated in EST sequencing, microsatellite marker validation and data analysis. V.O.C. participated in microsatellite marker validation and data analysis. F.L.E. participated in EST sequencing and data analysis. C.C.T. participated in EST sequencing. M.T.S.J. conceived the study and participated in EST sequencing. T.M. participated in EST sequencing. V.C.R.A. participated in microsatellite marker validation and data analysis. C.F.F. participated in microsatellite marker validation and data analysis. E.P.A. participated in microsatellite marker validation and data analysis. L.F.A.F. participated in EST sequence data analysis. N.F.M. participated in EST sequence data analysis. M.J.B.C. participated in northern blot analyses. F.C.B. conceived the study and participated in northern blot analyses. O.S. supervised and participated in EST sequence data analysis and computational searches for microsatellite identification and primer design. G.J.P.J. supervised and participated in EST sequence data analysis and computational searches for microsatellite identification and primer design, and editing of the manuscript. L.P. conducted *in vitro* bioassays. C.A. conceived the study and conducted *in vitro* bioassays. A.Y.C. conceived the study, prepared cDNA libraries and supervised microsatellite marker validation and data analysis. P.P. conceived the study, prepared cDNA libraries, participated in northern blot analysis and edited the manuscript. R.N.G.M. conceived the study, participated in EST sequencing and data analysis, and drafted the manuscript. All authors have contributed to, read and approved the final manuscript.

## Conflict of interest statement

None declared.
